# Non-collinear magnetic structure of manganese quadruple perovskite CdMn_7_O_12_

**DOI:** 10.1038/srep45939

**Published:** 2017-04-05

**Authors:** H. Guo, M. T. Fernández-Daz, L. Zhou, Y. Yin, Y. Long, A. C. Komarek

**Affiliations:** 1 Max-Planck-Institute for Chemical Physics of Solids, Nöthnitzer Str. 40, Dresden D-01187, Germany; 2 Institut Laue-Langewin (ILL), 71 avenue des Martyrs, F-38042, Grenoble Cedex 9, France; 3 Beijing National Laboratory for Condensed Matter Physics, Institute of Physics, Chinese Academy of Sciences, P. O. Box. 603, Beijing, 100190, China; 4 Collaborative Innovation Center of Quantum Matter, Beijing, 100190, China

## Abstract

We report on the magnetic structure of CdMn_7_O_12_ determined by powder neutron diffraction. We were able to measure the magnetic structure of this Cd containing and highly neutron absorbing material by optimizing the sample geometry and by blending the CdMn_7_O_12_ with Aluminum powder. Below its Néel temperature *T*_N1_ all magnetic reflections can be indexed by a single commensurate propagation vector ***k*** = (0, 0, 1). This is different to the case of CaMn_7_O_12_ where the propagation vector is incommensurate and where an in-plane helical magnetic structure has been found. We observe a commensurate non-collinear magnetic structure in CdMn_7_O_12_ with in-plane aligned magnetic moments resembling the ones in CaMn_7_O_12_. However, the commensurate propagation vector prevents the appearance of a helical magnetic structure in CdMn_7_O_12_. Finally, we also observe a third structural phase transition below ~60 K that can be attributed to phase separation.

The coexistence of ferroelectricity (FE) and magnetic ordering in multiferroic materials has attracted enormous attention in the past^1^. Especially the magnetoelectric coupling in these materials is of interest for possible technical applications. Multiferroics with spin induced ferroelectric properties seem to have the highest potential for sizeable magnetoelectric effects. Several possible mechanisms are able to induce FE by magnetic ordering, e.g. the inverse Dzyaloshinskii-Moriya interaction^2^, the exchange striction^3^ or the spin current^4^ and *d* − *p* hybridization effect^5^,^6^. The electric polarization (***P***) in these materials is, however, usually smaller than in conventional ferroelectrics and the magnetic transition temperature not very high^7^. Hence, the observation of a comparably large ***P*** of 2870 *μ*C/m^2^ in single crystalline CaMn_7_O_12_ has generated intense interest in this series of manganites^8^,^9^,^10^,^11^,^12^,^13^,^14^,^15^,^16^,^17^.

Multiferroic CaMn_7_O_12_^8^,^14^ belongs to the quadruple perovskite family with general formula (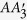)*B*_4_O_12_. The *A* site is 12-fold coordinated and *A*′ site is square-coordinated, while the *B* site is octahedrally coordinated. The *A*′ site is occupied by Mn^3+^ ions and the *B* site by mixed valent Mn^3.25+^ ions. Below ~440 K, CaMn_7_O_12_ undergoes a structural transition from cubic (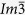) to rhombohedral (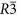)^18^,^19^. The *A*′ ions are located at Wyckoff position 9*e* in space group 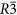, and the *B* site Mn^3+^ and Mn^4+^ ions are located at Wyckoff positions 9*d* and 3*b*. CaMn_7_O_12_ undergoes two successive magnetic transitions at *T*_N1_ ~ 90 K and *T*_N2_ ~ 48 K. In the temperature range *T*_N2_ < *T* < *T*_N1_ the magnetic structure is modulated along the *c*-direction with propagation vector ***k*** = (0, 0, 1.037)^8^. The magnetic structure below *T*_N2_ becomes more complicated due to the appearance of multiple propagation vectors^17^.

Except for CaMn_7_O_12_, the other quadruple perovskite manganites have to be synthesized under high pressure and high temperature conditions rendering the sample availability more difficult. A recent study on its analogue, CdMn_7_O_12_, has shown similar physical properties^11^. For example, CdMn_7_O_12_ also exhibits a structural transition at *T*_*s*1_ ~ 493 K and two successive magnetic transitions at *T*_N1_ = 88 K and *T*_N2_ = 33 K. Moreover, the magnetic transition at *T*_N1_ is robust against external magnetic fields, while *T*_N2_ can be gradually suppressed with applied magnetic field. The crystal structure of CdMn_7_O_12_ at room temperature is trigonal (space group 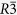). Around *T*_*s*2_ ~ 254 K a commensurate structural modulation 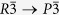 has been reported recently^15^.

In this paper, we report the magnetic structure of CdMn_7_O_12_ determined by powder neutron diffraction (PND). We focus on the temperature range *T*_N2_ < *T* < *T*_N1_, where the magnetic peaks can be indexed by a single propagation vector like in CaMn_7_O_12_. However, for CdMn_7_O_12_ the propagation vector amounts to ***k*** = (0, 0, 1) which is in contrast to CaMn_7_O_12_ where an incommensurate propagation vector along the *c*-direction has been observed. The magnetic structure of CdMn_7_O_12_ is non-collinear.

## Results and Discussion

**Figure 1:**
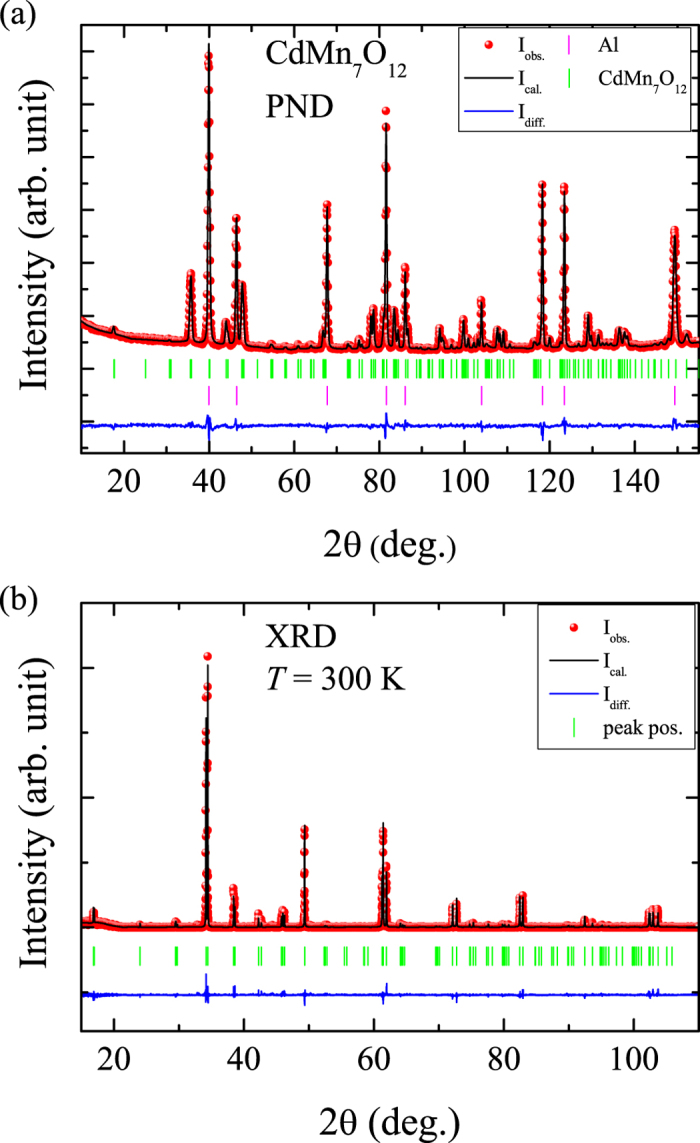
Rietveld fit of the room-temperature crystal structure of CdMn_7_O_12_ measured by (**a**) powder neutron diffraction at the D2B diffractometer with wavelength *λ* = 1.59 Å and (**b**) x-ray powder diffraction with wavelength *λ* = 1.5406 Å.

Figure 1 shows the Rietveld fit of the room temperature crystal structure measured both by PND and XRD (space group 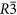). In Fig. 1(a), the few peaks of the Al powder did not interfere significantly with sample reflections and a reliable refinement of the crystal structure of CdMn_7_O_12_ could be obtained. The structural parameters are listed in Table 1. The refined volume fractions of Al and CdMn_7_O_12_ amount to 69.7(5)% and 30.3(2)% respectively.

Note, that indications for a structural transition 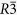 → 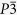 at *T*_*s*2 _~ 254 K have been reported recently^15^.

**Figure 2:**
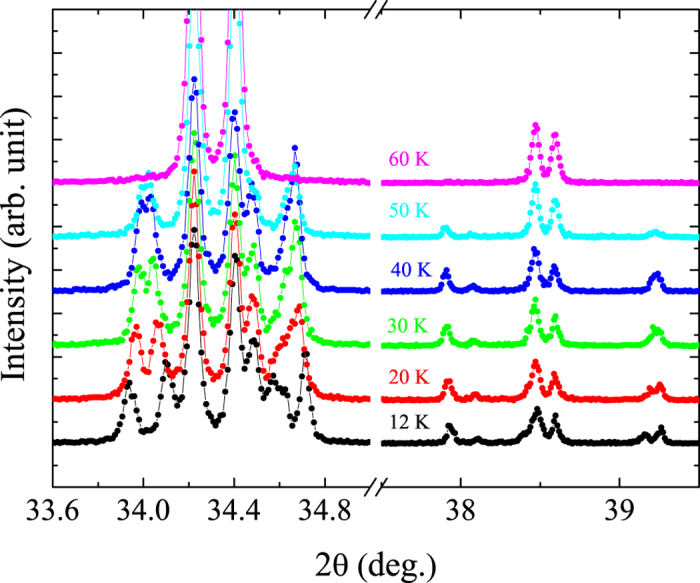
Temperature dependence of the XRD patterns measured at 60 K, 50 K, 40 K, 30 K, 20 K and 12 K. As can be seen, additional peaks appear below *T*_*s*3_ ~ 60 K. These additional peaks are indicative for phase separation and can be attributed to a second phase appearing below *T*_*s*3_. (The patterns are shifted for clarity).

We performed temperature dependant powder X-ray diffraction experiments down to 12 K and were able to observe an additional structural transition at *T*_*s*3_ ~ 60 K. Note, that there is a small anomaly at ~60 K in specific heat measurements at high magnetic fields of 9 T, whereas no obvious anomaly could be observed in zero field measurements^11^. Our measurements are indicative for the occurrence of phase separation below *T*_*s*3_ similar as observed in other transition metal oxides like SrCrO_3_^20^. Below *T*_*s*3_, the intensity of the main peaks related to the 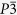 phase decreases and additional peaks appear which belong to a lower symmetry phase, see Fig. 2. This would be consistent with a scenario where phase separation occurs below *T*_*s*3_ and where only a certain volume fraction of the compound undergoes the structural transition (that might be enhanced by applying magnetic fields of 9 T). The origin of this structural transition requires further studies. Here, we focus mainly on the magnetic structure determination of CdMn_7_O_12_ slightly above or around *T*_*s*3_.

**Figure 3:**
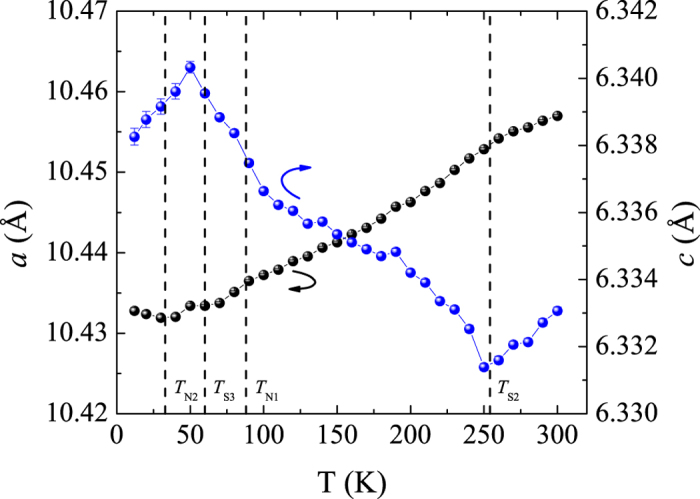
Temperature dependence of the lattice constant (space groups 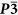 and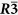) determined by x-ray powder diffraction.

We determined the lattice parameters of the 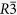 and 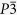 phases in the entire measured temperature range, see Fig. 3. The lattice parameters show an anisotropic thermal expansion. The *a* lattice constant decreases continuously with decreasing temperature until *T*_N2_. A small kink can be observed at *T*_*s*2_ while no obvious anomaly can be found at *T*_N1_. Then, below *T*_N2_, *a* starts to increase with decreasing temperature. On the other hand, the *c* lattice constant exhibits distinct anomalies at *T*_*s*2_ and also at 100 K and 50 K. This behaviour is similar to the one in CaMn_7_O_12_^21^. The temperatures of 100 K and 50 K are somewhat higher than the magnetic transition temperatures *T*_N1_ and *T*_N2_, suggesting that the magnetic transitions follow the structural ones.

**Figure 4:**
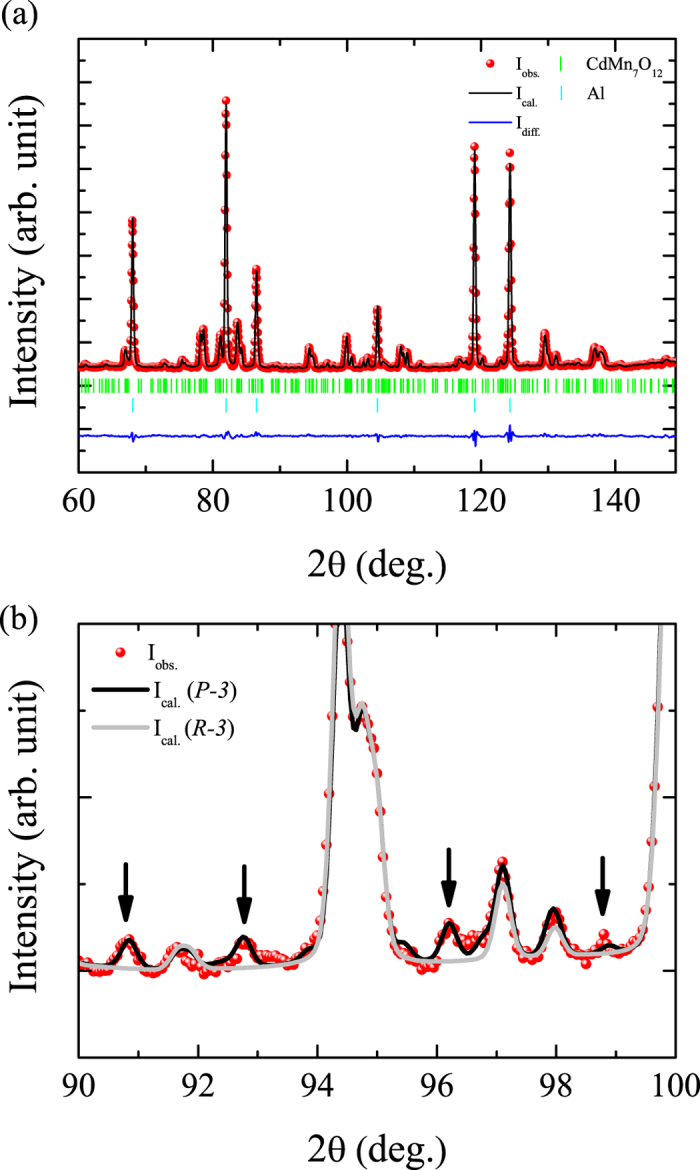
Rietveld fit of the powder neutron diffraction patterns measured at D2B diffractometer with wavelength *λ* = 1.59 Å at 40 K. (**a**) The nuclear structure is refined in the high 2*θ* range with space group 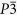. (**b**) Comparison of the pattern refined by using the space group 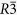 and 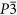. Arrows indicate the peaks which cannot be described by the space group 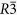.

We studied the crystal structure of CdMn_7_O_12_ at 40 K by means of powder neutron diffraction at the D2B diffractometer. The peak-splitting that has been observed in our high-resolution XRD measurements could not be resolved in our neutron measurements with less resolution. In Fig. 4(a) a refinement of the crystal structure with one single phase (space group 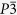) is shown. For comparison, the pattern refined with space group 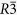 is also shown in Fig. 4(b). The additional peaks indicated by the arrows cannot be described by space group 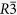. The structural parameters are listed in Table 2.

**Figure 5:**
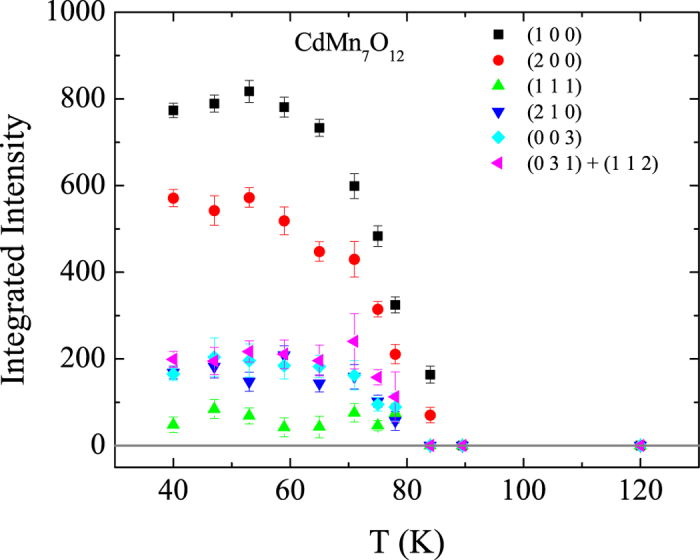
Temperature dependence of magnetic peaks observed in our PND patterns (*λ* = 2.52 Å).

**Figure 6:**
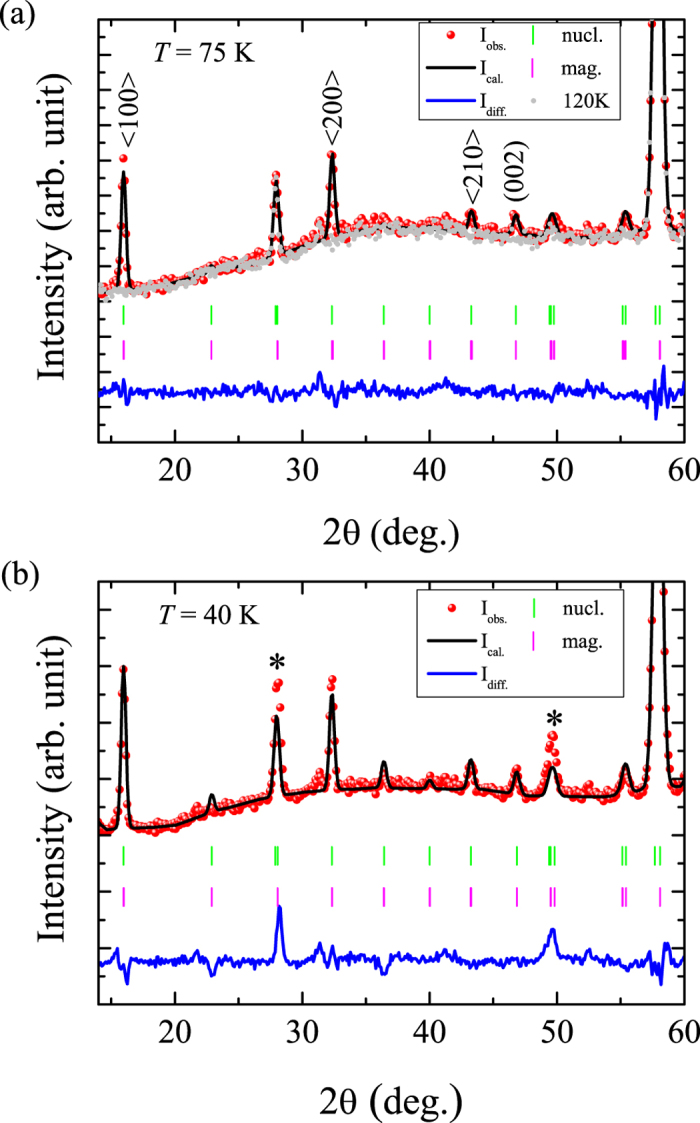
Rietveld refinement of the magnetic structure measured at the D1B diffractometer (*λ* = 2.52 Å). The magnetic structure at (**a**) 75 K (above *T*_*s*3_) and (**b**) 40 K (below *T*_*s*3_) can be described both by the IR Γ_3_. In (**a**), additionally, the 120 K data is shown as a reference. In (**b**) the two asterisks mark the nuclear peak positions that become broader and stronger due to the structural transition below *T*_*s*3_. However, the overall magnetic peak intensities can be still described by IR Γ_3_.

We next focus on the magnetic structure refinement. We have measured the magnetic structure of CdMn_7_O_12_ between 2 K and 120 K at the D1B diffractometer using an incident neutron wavelength of 2.52 Å. Only two peaks from the Aluminum powder appear in the entire diffraction pattern. Thus, the dilution with fine Aluminum powder does not cause any interference with magnetic reflections that appear below the Néel temperature of CdMn_7_O_12_. As can be seen in Fig. 5 all peaks that appear at low temperature and that can not be indexed with space group 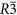 vanish at the Néel temperature of CdMn_7_O_12_. Hence, these reflections are magnetic in origin and can not be connected to the structural transition that takes place at 254 K. For *T*_N2_ < *T* < *T*_N1_ all magnetic peaks can be indexed with a single propagation vector ***k*** = (0, 0, 1), which is different from the incommensurate case in CaMn_7_O_12_. Below *T*_N2_ the magnetic structure is more complicated and multiple propagation vectors are necessary for describing the magnetic structure. Here, we will focus on the magnetic structure for *T*_N2_ < *T* < *T*_N1_ only. Due to the structural transition from 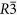 to 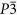, the three Mn sites split into six different sites below about 200 K. Instead of a refinement with that many independent magnetic moments and moment sizes we were also able to describe the magnetic neutron scattering intensities properly in a refinement based on irreducible representations for the high symmetry crystal structure 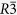 which is similar to a refinement in 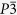 with certain constraints to the moments. Since the structural distortions below the structural phase transition 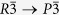 are negligible for the magnetic structure these constraints appear reasonable. Figure 6 shows the results of our refinements for temperatures of (a) 75 K and (b) 40 K.

The magnetic reducible representation Γ of site 9*e* and 9*d* is decomposed into three irreducible representations (IRs) with dimension one, as


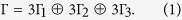


For site 3*b*, it is also decomposed into three IR with dimension one, as


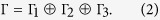


The basis vectors (*ψ*_*i*_) of the IRs are listed in Tables 3 and 4 for the 3*b* and for the 9*e*(9*d*) site respectively.

Since there are three different Mn sites in the unit cell, magnetic couplings between Mn ions at different sites require the Mn ions order according to the same IR in the first order approximation^22^. From Table 3, it can be seen that the IR Γ_1_ constrains the magnetic moments at site 3*b* along the *c*-direction, while for IR Γ_2_ and Γ_3_, the real and imaginary components are perpendicular to each other with the same length, thus, giving rise to a non-collinear magnetic structure with moments that have the same size and that are aligned within the *ab* plane. Earlier PND studies on CaMn_7_O_12_ suggested a ferrimagnetic ordering with magnetic moments along the *c*-axis^23^. However, the observation of the (0 0 2) peak at 2Θ ~ 47° is not indicative for this scenario based on IR Γ_1_. Also if *ab* components of Mn ions at 9*e* and 9*d* sites are included for the refinement based on IR Γ_1_ no satisfactory description of the measured data could be obtained. Since CdMn_7_O_12_ is a quite localized system, we also assume no modulations of the ordered moment sizes for the 9*e* and 9*d* sites. This can be realized by a linear combination of the basis vectors as follows:


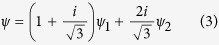


**Figure 7:**
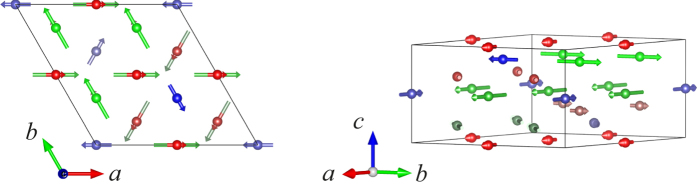
Magnetic structure of CdMn_7_O_12_ (T_N2_ < T < T_N1_) projected along the *c*-direction. The red, green and blue arrows represent the magnetic moment of Mn1, Mn2 and Mn3 at the crystallographic 9*e*, 9*d* and 3*b* sites respectively. The light and dark color represent Mn ions in different layers along the *c*-direction.

Thus, the IR Γ_2_ gives rise to a 120°-type noncollinear structure, while the magnetic moments are parallel to each other within the same layer for Γ_3_. The former will lead to the extinction of the (0 0 *l* + *k*_*z*_) reflection, which is not in agreement with our experimental observations (Fig. 6(a)). For these reasons the magnetic structure of CdMn_7_O_12_ can be described by the IR Γ_3_. The refinable parameters are the amplitudes of the moments at three sites and the phases between them. In our refinements the phase difference between Mn1/Mn2 and Mn3 turned out to be always close to *π*. Therefore, we fixed the phase to this value and only the amplitude of the moments was, finally, refined. The best refinement is shown in Fig. 6(a). As can be seen, all magnetic reflections can be described in a satisfactory manner; the magnetic *R* factor amounts to 22.5%. The magnetic structure is plotted in Fig. 7. Within the same sublattice the moments are parallel to each other within the same layer (same value of *z*) and rotate by 2*π*/3 and 4*π*/3 when going from one layer to the next according to the centering translations (2/3, 1/3, 1/3) and (1/3, 2/3, 2/3) respectively. The net moment in the unit cell is zero, which is consistent with the magnetization measurements between *T*_N1_ and *T*_N2_, showing linear curve of *M* vs. *H*. The magnetic structure resembles the one of CaMn_7_O_12_^8^,^24^. However, the magnetic structure of CaMn_7_O_12_ is incommensurate whereas the propagation vector is commensurate in the case of CdMn_7_O_12_.

**Figure 8:**
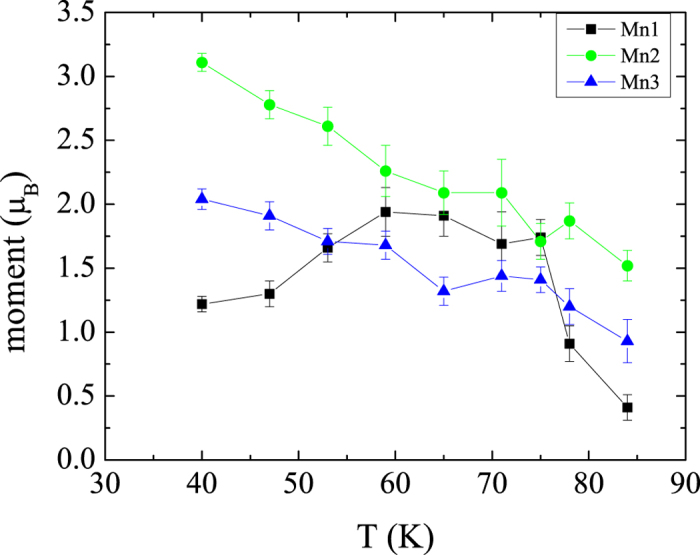
Temperature dependence of the magnetic moments for the distinct Mn sites.

The temperature dependence of the ordered magnetic moments for the distinct Mn sites is shown in Fig. 8. Below *TN*1, the ordered moment size starts to increase. Then, at *T*_*s*3_, the moments at the Mn1 site exhibit an anomalous decrease whereas the Mn2 and Mn3 sites continue their increase. Note, that the size of the magnetic moments of Mn^3+^ ions at the 9*e* (Mn1) and 9*d* (Mn2) sites (the 9*e* site is square coordinated, while the 9*d* site is octahedrally coordinated by O ions) is very similar for CaMn_7_O_12_ which is in contrast to the case of CdMn_7_O_12_.

In summary, the magnetic structure of CdMn_7_O_12_ below its Néel temperature has been determined by PND. We observe a non-collinear magnetic structure with Mn spins that are aligned within the *ab* plane. The magnetic propagation vector is commensurate with ***k*** = (0, 0, 1) which prevents the occurrence of a helical structure. This is different to the case of CaMn_7_O_12_.

## Methods

Polycrystalline samples of CdMn_7_O_12_ were synthesized by high pressure method as described elsewhere^11^.

Powder neutron diffraction (PND) measurements were performed at the D1B and D2B diffractometers at the Institut Laue-Langevin (ILL), France with a neutron wave length *λ* of 2.52 Å and 1.59 Å respectively. In order to reduce the absorption of neutrons by Cd nuclei, the CdMn_7_O_12_ powder was mixed with larger amount of Al powder and filled into the outer space of a hollow vanadium cylinder. This technique that we initially developed for SmFeO_3_^25^ enabled us to obtain excellent and fully evaluable neutron data of this highly neutron absorbing material (irrespective of the chosen neutron wavelength ~2.5 Å or ~1.6 Å). The PND patterns were analyzed by the Rietveld method using the Fullprof software.

In addition, temperature dependent x-ray diffraction (XRD) measurements were also performed on a Bruker D8 Discover A25 diffractometer using Cu 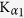 radiation. A closed cycle helium cryostat (*Phenix* of *Oxford Cryosystems*) was used for cooling the flat plate powder sample.

## Additional Information

**How to cite this article**: Guo, H. *et al*. Non-collinear magnetic structure of manganese quadruple perovskite CdMn_7_O_12_. *Sci. Rep.* **7**, 45939; doi: 10.1038/srep45939 (2017).

**Publisher's note:** Springer Nature remains neutral with regard to jurisdictional claims in published maps and institutional affiliations.

## Figures and Tables

**Table 1:** Atomic position of CdMn_7_O_12_ at room-temperature obtained from Rietveld refinement of the PND pattern measured at D2B.

atom	x	y	z	B_*iso*_
Cd1(3a)	0	0	0	1.36(18)
Mn1(9e)	1/2	0	0	0.62(5)
Mn2(9d)	1/2	0	1/2	0.62(5)
Mn3(3b)	0	0	1/2	0.62(5)
O1(18f)	0.2264(4)	0.2789(4)	0.0822(4)	1.02(2)
O2(18f)	0.3430(4)	0.5235(3)	0.3467(7)	1.02(2)

The reliability factor is *R*_Bragg_ = 8.88%, *χ*^2^ = 2.76. The space group is 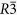, lattice constant *a* = 10.4306(1) Å, *c* = 6.3184(1) Å.

**Table 2:** Atomic positions of CdMn_7_O_12_ at 40 K as obtained from Rietveld refinement of the PND pattern measured at the D2B diffractometer.

atom	x	y	z	B_*iso*_
Cd1(1a)	0	0	0	0.27(14)
Cd2(2d)	0.3333	0.6667	0.6784(38)	0.27(14)
Mn1(3e)	1/2	0	0	0.20(6)
Mn2(3f)	1/2	0	1/2	0.20(6)
Mn3(1b)	0	0	1/2	0.20(6)
Mn4(2d)	0.3333	0.6667	0.1471(29)	0.20(6)
Mn5(6g)	0.3357(17)	0.1608(16)	0.6529(21)	0.20(6)
Mn6(6g)	0.1653(15)	0.8216(16)	0.8333(24)	0.20(6)
O1(6g)	0.2366(17)	0.1608(16)	0.6529(16)	0.92(3)
O2(6g)	0.3486(14)	0.5223(16)	0.3486(20)	0.92(3)
O3(6g)	0.0687(10)	0.6148(14)	0.7536(20)	0.92(3)
O4(6g)	0.2815(16)	0.8988(13)	0.5815(23)	0.92(3)
O5(6g)	0.3192(16)	0.8098(15)	1.0006(18)	0.92(3)
O6(6g)	0.1461(14)	0.1458(13)	0.6914(17)	0.92(3)

The reliability factor amounts to *R*_Bragg_ = 7.49% with *χ*^2^ = 8.66. We used one single phase with space group 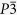. The lattice constants amount to *a* = 10.4064(1) Å, *c* = 6.3250(1) Å.

**Table 3:** Basis vectors of the irreducible representations of the space group 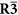 for sites 3b with propagation vector *k* = (0, 0, 1).

Mn3 (3*b*)			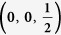
Γ_1_	*ψ*_1_	Re	(0, 0, 1)
Γ_2_	*ψ*_1_	Re	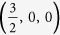
		Im	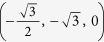
Γ_3_	*ψ*_1_	Re	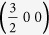
		Im	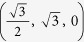

**Table 4:** Basis vectors of the irreducible representations of the space group 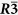 for sites 9*e* and 9*d* with propagation vector *k* = (0, 0, 1).

Mn1 (9*e*)			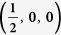	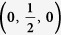	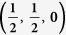
Mn2 (9*d*)			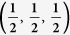	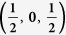	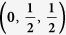
Γ_1_	*ψ*_1_	Re	(1, 0, 0)	(0, 1, 0)	(−1, −1, 0)
	*ψ*_2_	Re	(0, 1, 0)	(−1, −1, 0)	(1, 0, 0)
	*ψ*_3_	Re	(0, 0, 1)	(0, 0, 1)	(0, 0, 1)
Γ_2_	*ψ*_1_	Re	(1, 0, 0)	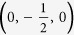	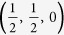
		Im	(0, 0, 0)	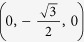	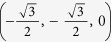
	*ψ*_2_	Re	(0, 1, 0)	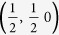	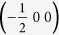
		Im	(0, 0, 0)	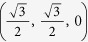	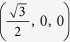
	*ψ*_3_	Re	(0, 0, 1)	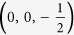	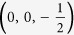
		Im	(0, 0, 0)	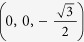	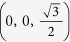
Γ_3_	*ψ*_1_	Re	(1, 0, 0)	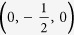	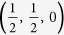
		Im	(0, 0, 0)	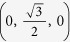	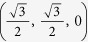
	*ψ*_2_	Re	(0, 1, 0)	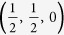	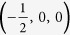
		Im	(0, 0, 0)	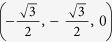	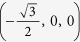
	*ψ*_3_	Re	(0, 0, 1)	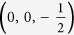	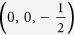
		Im	(0, 0, 0)	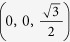	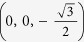
