# Corrigendum: Non-collinear magnetic structure of manganese quadruple perovskite CdMn_7_O_12_

**DOI:** 10.1038/srep46775

**Published:** 2017-05-04

**Authors:** H. Guo, M. T. Fernández-Díaz, L. Zhou, Y. Yin, Y. Long, A. C. Komarek

Scientific Reports 7: Article number: 4593910.1038/srep45939; published online: 04 05 2017; updated: 05 04 2017

The original version of this Article contained an error in the spelling of the author M. T. Fernández-Díaz, which was incorrectly given as M. T. Fernández-Daz. This error has now been corrected in the PDF and HTML versions of the Article.

